# A Content-Analysis Based Literature Review in Blockchain Adoption within Food Supply Chain

**DOI:** 10.3390/ijerph17051784

**Published:** 2020-03-09

**Authors:** Jiang Duan, Chen Zhang, Yu Gong, Steve Brown, Zhi Li

**Affiliations:** 1 Blockchain Research Center of China, Southwestern University of Finance and Economics, Chengdu 611130, China; duanj_t@swufe.edu.cn; 2 Southampton Business School, University of Southampton, Highfield, Southampton SO17 1BJ, UK; y.gong@soton.ac.uk (Y.G.); steve.brown@soton.ac.uk (S.B.); 3 School of Electromechanical Engineering, Guangdong University of Technology, Guangzhou 510006, China; piersli@foxmail.com

**Keywords:** blockchain, food supply chain

## Abstract

According to the World Health Organization (WHO), one out of 10 people get sick from eating contaminated food. Complex food production process and globalization make food supply chain more delicate. Many technologies have been investigated in recent years to address food insecurity and achieve efficiency in dealing with food recalls. One of the most promising technologies is Blockchain, which has already been used successfully in financial aspects, such as bitcoin, and it is attracting interests from food supply chain organizations. As blockchain has characteristics, such as decentralization, security, immutability, smart contract, it is therefore expected to improve sustainable food supply chain management and food traceability. This paper applies a content-analysis based literature review in blockchain adoption within food supply chain. We propose four benefits. Blockchain can help to improve food traceability, information transparency, and recall efficiency; it can also be combined with Internet of things (IoT) to achieve better efficiency. We also propose five potential challenges, including lack of deeper understanding of blockchain, technology difficulties, raw data manipulation, difficulties of getting all stakeholders on board, and the deficiency of regulations.

## 1. Introduction

Food systems are complex and keep changing over time [1,2] Nowadays, customers have an increasing demand for food quality, safety, and nutrition, rather than just quantity. Although hunger might seem not to be an issue for most people anymore, food crisis is still ranked as the seventh risk in terms of impact by the World Economic Forum in 2018 [3]. Moreover, globalisation and outsourcing make the food system even more complex with more suppliers and companies involved [4,5,6,7]. Every continent has suffered serious foodborne disease outbreaks and they have been amplified by globalisation in the past decade [7]. The longer geographical distance between the producers and consumers of food supply chains has also created a challenge in maintaining food quality and achieving fast food recall when necessary.

According to the World Health Organization [8], almost one out of 10 people globally suffer sickness due to foodborne disease each year. Food hazards can cause over 200 diseases by bacteria, chemical, and other contaminations; and, the foodborne and waterborne diarrhoeal diseases can kill about two-million people annually [8]. Some of the most well-known food scandals in history include Sanlu milk scandal in China (2008), the Enterohemorrhagic Escherichia coli outbreak in Germany due to contaminated fenugreek sprouts (2011), the horse meat scandal in the UK (2013), and the outbreak of E. coli of Romaine lettuce in America (2018). More recently, several foodborne disease outbreaks were reported. For example, an outbreak of Salmonella Newport illnesses that was linked to frozen ground tuna in America caused 13 people to fell ill [9], Salmonella-infected British eggs poisoned 45 people [10], in the Netherlands, and 12 people felt sick due to the Listeria monocytogenes infected meat [11].

In America, approximately 48 million people get sick and 3,000 of them die annually due to foodborne diseases [12]. In 2018, there were 1,935 food recalls reported according to US Food and Drug Administration [9]. More than half of the food recalls were due to operational mistakes, including contamination, mislabelling, undeclared ingredient, biological causes, etc. [13]. Customers have growing concerns of food security and quality. From the food safety survey conducted in 2018, more than two-thirds (68.3%) of participants were worried about food fraud problems [14]. A meat inspection petition that was signed by more than 216,000 people was submitted to the UK food standards Agency [15]. Food security is considered to be a shared responsibility for all of the stakeholders, including food producers, food retailers, related governments, and customers [7,16].

Supply chain management is a key vehicle for helping to address food insecurity and contribute to public health issues. Within a complex food supply chain, the efficient traceability system can make significant contribution in food recall and public health. It can help to isolate certain products and ingredients from the root of the problem in a faster speed to prevent further loss. Traditionally, the traceability system largely relied upon paper-based systems or internal computer systems [16]. Paper recording can be time consuming and cause errors. Internal traceability can be unusable for other companies and cause difficulties for stakeholders’ integration. Technologies recently showed popular trends, such as Radio Frequency Identification (RFID), barcodes, smart tags, Wireless Sensor Network (WSN), and DNA based techniques. Technological innovations can provide a more efficient way to record and exchange information. Most recently, blockchain has drawn significant attention and presents a promising solution to the food traceability issues [17].

Blockchain technology is one of the promising technologies, which is believed to revolutionise the modern food supply chain [18,19]. It is a decentralized platform that not only allows peer-to-peer direct transaction that eliminates middlemen, but also validates information by cryptography and records history permanently. A few researchers suggest that it can improve supply chain efficiency and address certain issues, such as information inequality and food recall inefficiency, etc. [19,20,21].

Previous researches have introduced a few integrations of blockchain and food supply chain, with the application of Internet of Things (IoT) [17,22], by applying case studies [23], or Survey methods [24]. These studies presented some benefits, such as improving traceability efficiency, improving supply chain transparency, while there are also challenges, including scalability, lack of legislation, and immature technology [21]. However, few literature reviews have been carried out to explore this topic in a systematic manner, especially with a management focus. Therefore, this paper aims to fill this gap. Thus, the primary aim of this paper is to investigate how blockchain has been used in the food supply chain area, and how it can help to address food security issues. Therefore, this paper answers the following research questions:

- **Question 1:** What research has been carried out on blockchain adoption in food supply chain management?
- **Question 2:** What benefits can blockchain bring to the food supply chain?
- **Question 3:** What are the challenges of blockchain adoption in food supply chain management?

In order to answer these questions and enable achieve the aim of the study, we will collect and summarise related papers, and provide a deeper analysis of the literature. More specifically, this paper applies the content-analysis based literature review methodology to support this analysis.

The remainder of this paper is organized, as follows. We first introduce literature review to provide some background information about the key concepts followed by our research methodology. We then present and discuss the findings and, finally, conclude and discuss future research direction.

## 2. Literature Review

This section provides a brief review of the relevant concepts, including food supply chain, Blockchain, and traceability, to provide some background information as the basis for this study.

### 2.1. Food Supply Chain

Food supply chain is defined by Folkerts and Koehorse [25] (p. 11), as “a set of interdependent companies that work closely together to manage the flow of goods and services along the value-added chain of agricultural and food products, in order to realize superior customer value at the lowest possible costs”. When compared to other industries, food production takes place in more vulnerable value chains, which requires more attention over handling processes, such as producing and storing [16,26,27]. In addition, food has the natural feature of changing in quality all the time, which makes ensuring food safety and quality a challenge [16]. Outer environments, such as temperature and transport, can also affect food products’ quality and freshness. Processed food with longer shelf lives might have significantly complex producing procedures with a mixture of multiple ingredients. The complex food production also means higher risks of product failure and requires extra attention on the raw material quality and production process [6,26,28]. Food products failure includes food borne disease, food poisoning, low quality food, counterfeit products, or mislabelling and undeclared ingredients after production. Every step and every supplier in a food supply chain matters to the final food products. Therefore, food supply chain requires higher efficiency and closer partner collaboration to maintain the value chain and eliminate products failure.

Modern food supply chain is centralized, which heavily relies on the central powers to control information flow. The centralization can threaten the supply chain transparency, which causes information inequality and trust issues [17,22]. Companies can choose to open up selected information that is beneficial to its own brand image [29]. On the other side, companies can also hide information, so that customers can only know what food companies and governments want them to know. Centralized supply chain also means the vulnerability for being a target of bribery [17]. Therefore, a single failure can lead to the disruption of the whole supply chain network [17]. For certain products, such as organic, kosher products, vegan food or green products, it is even harder for consumers to know the authenticity if there is no evidence of customers becoming sick as a result of consuming the products. Therefore, customers feel more threatened by the food scandals and demand more knowledge of the products before purchasing. Deeper concerns and lack of trust in the food industry and food quality still remain, even where laws and policies have been published.

### 2.2. Blockchain

Blockchain has recently gained considerable research attention for addressing food supply chain issues. Nakamoto introduced the concept of the decentralized peer-to-peer ledger in 2008. It has been successfully applied in financial areas, such as Bitcoin, and it now triggers huge interest in multiple areas, including food supply chain, property, voting, etc. Blockchain technology can be defined as “a shared, immutable ledger for recording transactions, tracking assets and building trust” [30]. The fundamental technology of blockchain has a few main features that can bring significant benefits once used properly: decentralization, immutability, security, and smart contract.

#### 2.2.1. Decentralization

Different from traditional transactions that need to be approved by central authorities, decentralization eliminates the central powers and addresses information inequality by allowing for direct transaction between the users. It ensures that every authorized user has equal power within a network. Users help each other to validate transactions, to keep copies of records, and have the same power to access history anytime [19,31,32]. In the food supply chain, from the raw material suppliers to customers, products information can be recorded along the whole supply chain. Multiple stakeholders save the copies of records, which can be retrieved on demand anytime [33]. For example, the end users, consumers, can obtain the detailed information on the products include authenticity and origins, etc. Producers can also monitor their suppliers to make sure that raw material quality meet requirements. Therefore, a decentralised supply chain can help to eliminate information inequality and build trust.

#### 2.2.2. Security

The blockchain consensus algorithm can achieve data security. One of the consensus mechanisms is Proof of Work (PoW), which requires that all transactions, and is validated by other users [34]. Users have to define computer calculation to approve a transaction and add data into the database. When decentralization eliminates central power on the network, it also prevents a supply chain from breaking down, because a single point failure will not lead to the failure of the whole network, which can reduce the chance of hacking. Technically, hacking can only be achieved when the majority of the users are taken over, which will take a considerable amount of energy/time [34]. Therefore, the more complicated blockchain network is with more users, the more difficult it is for the hacking behaviour to happen. When applied to the food supply chain, blockchain can keep records and data safe, and eliminates the risks of hacking and data stealing.

#### 2.2.3. Immutability

After allowing authorized users to have the same power to upload and check information, blockchain also ensures the records’ originality and authenticity. This means historic data cannot be altered without warning other users. Therefore the immutability feature can minimize the human intervention on records. This feature is especially useful during food recall, which can prevent any related stakeholders change history and escape from responsibilities [19,33]. It is powerful proof for investigating the possibilities of food crisis. It allows companies to trace back along the supply chain and isolate certain ingredients from specific suppliers more efficiently [23,35]. It also acts as evidence for containing products, such as organic food, halal food, fair trade food, etc., which allows for customers to buy with confidence. However, immutability cannot always guarantee the raw information authenticity, and the initial data has to be correct from the beginning. With the increase of transactions, blockchain can be considered as a strategic tool to encourage food supply chain stakeholders to take responsibilities and provide/record quality information [20,23].

#### 2.2.4. Smart Contract

Smart contract is another important feature of blockchain, which is a digitalized contract and operates automatically once certain agreements are met [34]. The use of smart contact can significantly speed up the transactions and enhance trust [35,36,37]. For instance, payment can automatically be sent to producers once products arrive at the warehouse. The programmed contract can save paperwork, accelerate processing time, and minimize human labor efforts when compared to the traditional supply chain [21,23]. For example, in 2014, Maersk found that over 30 people and organizations got involved when shipping a container of roses and avocado from Kenya to Netherlands [38]. It also took 34 days, including 10 days of documents processing to complete a whole shipping activity, not considering missing papers causing delays and time extension into account [38]. No single users can make changes on smart contract as it is based on the agreement of all partners. In other words, it can replace “the letter of credit” and protect the partnerships.

Combining all of these features, blockchain can eliminate the risks of transactions in a lack of trust environment, increase supply chain visibility and transparency, improve efficiency, and protect every stakeholder’s benefits. In this case, blockchain can achieve maximum digitalization, save processing time, increase efficiency, and reduce unnecessary costs [39].

### 2.3. Traceability

Traceability has many definitions so far, the earliest definition was by International Organization for standardization in 1994 [40]: “the ability to trace the history, application or location of an entity by means of recorded identifications”. The Codex Alimentarius Commission defines Traceability as “the ability to follow the movement of a food through specified stage(s) of production, processing and distribution” [41]. Bosona and Gebresenbet [42] (p. 35) proposed the definition as: “food traceability is part of logistics management that capture, store, and transmit adequate information about a food, feed, food-producing is correct animal or substance at all stages in the food supply chain so that the product can be checked for safety and quality control, traced upward, and tracked downward at any time required”.

Many researchers have proposed few benefits on food traceability [16,43,44], which can be summarized into four types: improve efficiencies (increasing food safety, improving operational efficiencies, enhancing brand reputation); meet stakeholder demand (meeting stakeholder requirements and ensuring food authenticity); meet regulations requirements (meeting legal requirements); and, achieve global alignment (supporting global standardization and conservation of natural resources).

Good traceability provides precise recording of products movements, which allows companies to have a clearer view of the supply chain, make better decisions, and avoid potential quality risks [44]. The ability of tracing products backward and forward along the supply chain can improve the speed of isolating and finding certain products from certain suppliers, which makes quality checks and product recalls more efficient. By demonstrating the resources and products flow, customers have better knowledge and trust in the buying products.

Therefore, traceability is considered as an added value to the food products [43,45]. In addition, the traceability system can be a marketing tool to attract more customers and enhance customer loyalty. The records keeping are also a point of verifications for companies to ensure their suppliers provide quality products [16]. Therefore, an efficient traceability system can be used as a strategic tool to build trust between partners [43]. Following the food hazards and customer demand, governments and non-government organizations have also published policies to encourage and pressure food companies to develop their traceability systems for food safety and quality purposes. For example, European Commission (EC) Food Law Regulation 178/2002 was developed to ensure the food traceability requirements and stated that food must be traceable at all stages [44].

Many government departments or non-profit organizations were also built to help with food safety, such as FDA in America, Food Standards Agency in the UK, State Food and Drug Administration in China, etc. The global supply chains are more complex with different policies to adapt in different areas, and give global companies more pressure for traceability. By applying blockchain, globalized standardization can be adaptable for all countries and regions, and they can save companies from duplicative works [44]. For sustainability purposes, traceability is also a way to monitor environmental impacts, therefore, encouraging companies to be more sustainable.

Although traceability is found to be important and necessary, it can differentiate food companies from success and failure during food recall, by accelerating the recall speed and saving unnecessary costs [43]. Traditionally, product recall can take up to weeks or months due to the complicated and inefficient procedures, especially for processed food. According to FDA, it takes, on average, 57 days for a recall, sometimes it can take up to 10 months [46,47]. The current traceability systems are largely paper-based or by private databases [16], which is even harder to achieve efficient target recall [34]. The slower the products recall, the more likely the harm to public health [34]. It can also lead to deep concerns of food safety and damage a company’s brand image. Therefore, an efficiency traceability system can be a “life saver” for both customers and companies.

Blockchain was found to be a choice of solution for achieving efficient traceability in the food supply chain [22] utilized one of the earliest researches of blockchain in food traceability. In this paper, the author proposed a conceptual framework that integrates blockchain and IoT, and analyses the benefits, including improving efficiency and transparency. There are also many pilot studies that provide the practical implication. One of the well-known blockchain pilot examples was run by Walmart and IBM to trace mango [32]. When compared with the traditional traceability system, mango reduced the tracing time from nearly seven days to 2.2 s by using blockchain.

## 3. Methodology

This research adopts a literature review method in order to answer the research questions. There are several review papers on blockchain and supply chain management; however, none of them have a specific focus on food supply chain with a management focus [19,21,31,35,48,49]. These papers provide the foundation for this research.

Fink [50] (p. 3) defined literature review as “a systematic, explicit, and reproducible design for identifying, evaluating, and interpreting the existing body of recorded documents”. Tranfield et al. [51] (p. 208) also suggested that literature review could be used “to map and assess the existing intellectual territory, and to specify a research question to develop the existing body of knowledge further”. This paper adopts a content-analysis based literature review method to analyze the literature on the application of blockchain in food supply chain management. Systematic review, according to Tranfield et al. [51] (p. 209, p. 220), aims to identify “key scientific contributions”, to reduce bias and “provide collective insight” to a field. Content analysis, as Seuring and Gold [52] suggested, can be used to generate reliable findings due to the structured and consistent procedure. A few researchers have conducted a content-based literature review method in the field of supply chain management [52,53]. This paper not only thoroughly reviews blockchain, but also considers practical applications in food supply chain management. It aims to provide a more precise and integrated understanding of blockchain and its influences in food supply chain management.

We apply a six-stage refinement process that was suggested by Durach et al. [54]: define research question, set inclusion and exclusion criteria, determine searching databases, apply criteria, synthesize relevant literatures, and report findings. Research questions were provided in the introduction. In order to have a wide coverage, the research keywords are: blockchain, food supply chain. Therefore, the journal articles and conference papers about blockchain applications in the food supply chain can be found and reviewed. Web of science, Scopus, and Ebsco are the three online databases that were applied to search for relevant academic literature, as the three databases have a wide range of resources, and they have been extensively used in supply chain management research. Peer-reviewed journal articles are seen as a way of high-quality communication between research fellows. However, in this paper, other resources, such as conference papers, grey papers, such as consulting reports, and third party reports that can provide more updated information, are also considered due to the early stage of blockchain and the limited published articles. The initial search came out with 57 results in the three databases.

**Figure 1:**
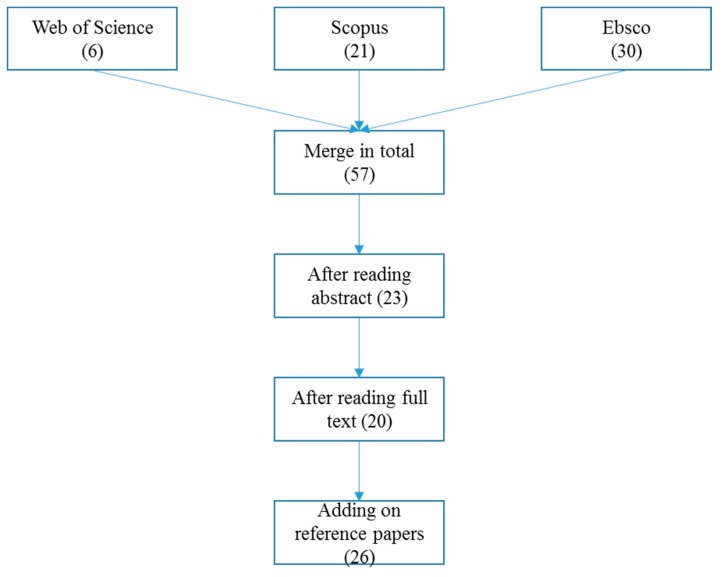
Paper selection process.

Removing the duplicated papers and applying inclusion and exclusion criteria (Table 1), the number of useful papers was reduced to 26 in the final process (Figure 1). The final 26 papers were categorized and evaluated by the content analysis method, which is a systematic and objective research method that have been used to quantify phenomena, documents, or communications [52].

Descriptive analysis is the first insight into all of the papers, to provide basic information of the selected papers. Among the selected papers, the earliest paper (one out of 26) was released in 2016, and nine papers in 2017, and 16 papers were from 2018, due to the young age of technology. The time trend shows that blockchain is gaining increasing attention and interest in the supply chain area. This also explains why 13 out of 26 papers are technology and innovation related conference papers. Although all of the papers are focused on the food supply chain, the food categories are slightly different. Most of the papers focused on agri-food supply chain in general [17,22,28,55,56,57,58,59,60,61]. A few papers used more specific food supply chains, such as Halal supply chain [62], tilapia supply chain [63], and rice supply chain [64]. Papers mainly introduced blockchain and demonstrate its potential by using conceptual framework (13 out of 26), and then pilot cases (seven out of 26), three theory papers (three out of 26), one survey (one out of 26), and two systematic literature analyses (two out of 26), respectively.

All of the papers were read thoroughly and highlighted by the different categories of benefits and challenges (Table 2). The benefits can be divided into four categories: transparency, information authenticity, efficiency, and sustainability; the challenges are: lack of understanding, immature technology, stakeholder cooperation, trade secrets, and raw data manipulation. By listing and dividing papers, as in Table 2, the brief content and highlights of every paper can be shown more clearly.

## 4. Finding and Discussion

Many studies pointed out the problems in current food supply chain, including inefficient traceability, information asymmetry, information fraud, poor supply chain management, etc. In this case, blockchain is supposed to be a feasible solution for the problems that are mentioned above. The following parts are the five benefits that have been summarized from the 26 papers (details in Table 2).

### 4.1. Benefits

#### 4.1.1. Blockchain Improves Food Traceability

Different food companies have different traceability systems, either by paper or by computer system. However, food supply chain can be very complicated with multiple supplies horizontally and vertically due to the complex feature of the food products [72]. Therefore, manual traceability system, such as paper records, can be very time consuming or easily cause errors. While some companies’ traceability database can be too private to be applicable for the other stakeholders, which can cause lack of stakeholder cooperation or a lack of supervision within a supply chain [59]. The complex food supply chain also means higher risks of recall (mislabelling, chemical contamination, low quality of raw material, and food additives) and higher requirements on stakeholders’ collaboration, single ingredient caused food recall can take up to many months to address [28,32]. According to Yiannas [32] it can cost up to $93 billion to recall products, due to an inability to trace the root cause of outbreak. Blockchain is believed to improve the traceability efficiency and enhance trust during food recall.

Galvez et al. [67], who analysed the potential of its uses in food traceability and authenticity, also suggested that blockchain is a powerful tool to avoid food fraud and improve traceability efficiency, including time and costs saving, risks reducing, and increasing trust. Similarly, Caro et al. [55] built two traceability systems that were based on combination of blockchain and IoT on Ethereum and Hyperleger Sawtooth, respectively, and confirmed the ability of blockchain on providing transparency and auditability. Walmart and IBM conducted a pilot study of the blockchain based traceability system in 2016. The two companies worked together to trace mangoes from a store to the farm. By current traceability system, it took almost seven days to collect all of the information of mango movements, which required every stakeholder to contact each other to get to know the required details [32]. By blockchain, the time to contact and wait for response from other stakeholders can be eliminated. The movements of mangoes are recorded by each stakeholder along the supply chain, and are ready to be checked anytime. The trace time reduced from nearly seven days to 2.2 s by blockchain [32]. AgriDigital and CBH group also carried out a pilot study in the Australian grain industry and found that the blockchain network allowed for better traceability [56].

#### 4.1.2. Blockchain Improves Food Supply Chain Transparency

In the current supply chain, big food brands normally choose to open partially selected information to the public and aim to benefit the companies themselves, which can cause customers to not have enough knowledge of products and company details of their suppliers [27]. As Reyna et al. [34] proposed that, insufficient information could impact food security. For certain products that have specific requirements, such as halal food, transparency of the supply chain is significantly necessary to ensure products quality and keep customer trust [62]. Although government and other authorities have published policies and make regular checks on food quality in many cases, the authority’s power can be the target of bribery, and contribute to cover up for big brand companies. For example, the Sanlu milk scandal did not become exposed in the first place because the company managers and local authorities hid the scandal [73]. Even though companies can release certain information based on requirements, it is easy to change information or erase history to escape from responsibilities or hide the truth [17,22,55,74]. Thus, information credibility and trust are hard to achieve by centralized supply chains, where transparency and visibility remain low [59].

Therefore, one of the main features of blockchain is decentralization, which allows for authorized users to make transactions and to access history directly without central power intervention. Every registered valid user has the same power to examine a transaction, and have a copy of history [19]. This feature can eliminate any large powers over the information flow, address information asymmetry between stakeholders, and provide transparency along the supply chain. Meanwhile, once data are updated onto the blockchain, the recordings become permanent. The immutability feature can be achieved by running the blockchain mining process [17]. Once the majority of miners/users vote to validate certain transactions, the transaction data stay stored, and they can never be changed without notifying all of the users [17]. Thus, the history of the products movements in a supply chain can be retrieved and checked any time whenever needed without worrying its being tempered. In addition, product information includes its movement and certain certification can be digitalized and updated, which allows for permissioned users to access anytime [32]. For food products, the verifications are necessary for proving companies’ eligibility for producing or selling. The digitalization of records and documents not only can save time from manual paper check, but also eliminate the risks from data manipulation and errors [56]. In 2016, Walmart and Tsinghua University tracked pork in China from-farm-to-fork [32]. The finding showed the ability of blockchain on improving information authenticity, reducing information errors, and gaining trust.

#### 4.1.3. Blockchain Can Be Combined with IoT Devices

IoT includes RFID (Radio-Frequency Identification), GPS (Global Positioning system), GIS (Geographic Information System), WSN (Wireless sensor network), etc., is an intelligent, reliable, and high-speed information network that connects objects. Instead of manual recording, information, such as temperature and humidity, can be automatically captured by IoT sensors. This real-time information capturing ability is especially important for frozen and fresh food products, as the quality is closely related to the external environment [22,65]. The automation by IoT can increase the efficiency of monitoring and capturing information, and reduce the manual errors [17,22,28,75]. However, there are also a few challenges of IoT deployments including data confidentiality, vulnerability, data integrity, and stakeholder’s privacy [34,55,75,76,77]. Therefore, protection and security are important when using IoT devices in supply chains, which can be developed by combining with blockchain protocol [19,34,35,39,75,76].

Many studies have combined blockchain technology with IoT and suggested that blockchain can help to manage IoT and make supply chain more efficient [17,22,28,63,67,68,75]. Rejeb [75] made six propositions on the combination of blockchain and IoT, the scalability, security, auditing, efficiency, interoperability, and the quality of IoT solutions can be improved. Tian [17,22] built an agri-food traceability system based on RFID tags and blockchain to deliver real-time information of food products. Lin et al. [28] also proposed a blockchain and IoT based agriculture system and agreed that the new system is trusted and self-organized without human intervention.

In practice, the Accenture traceability report listed a few blockchain pilot studies that have been incorporated with IoT, such as WWF while using smart tagging combined with blockchain to prevent illegal tuna fishing in Fiji; Belagricola uses IoT and smart contact to track grains and ensure the quality [68]. By combining with smart contract, once anything goes wrong, such as losing temperature control, the digitalized program can be automatically triggered and send registered users warnings, which can prevent further damage [17,55,65]. From the production to retailing, the integration of the two technologies allows for data collection and transferring without human intervention and ensures food quality and safety.

#### 4.1.4. Blockchain Can Improve the Efficiency of Food Recall

By using blockchain, the food supply chain is found to be more sustainable by operating more efficiently and targeted food recall. When products information is updated on blockchain in near real-time speed, stakeholders can have more knowledge of products flow and react to situations quicker. For instance, Walmart realized that fresh import products, such as mangoes, could wait to be checked up to four days in the country border [32]. In this case, Walmart can follow up the products movements, accelerate the products checking process, and give products greater shelf-live. The improvement of information transparency can improve the supply chain efficiency and eliminate unnecessary products wastes.

Meanwhile, the untargeted food recall is also one of the major causes of food wastes. Food products tend to be complicated with mixture of many ingredients. Single ingredient contamination caused food recall to be complicated and time consuming, which requires the traceability system to be extremely efficient. By blockchain-based traceability, it is possible to retrieve needed information, isolate products from certain suppliers, and narrow down the products recall range. Meanwhile, companies can also make more accurate customer demand forecasting based on the point of sales data, depending on the information [78]. Zhao et al. [21] also pointed out that applying blockchain technology could lead to sustainable water management by recordkeeping in blockchain platform based water trading.

### 4.2. Challenges

Blockchain sounds like a promising technology that might revolutionize food supply chain, improve efficiency, and eliminate risks. More pilot studies have been tested and received positive feedbacks [32,56]. However, it is an undeniable fact that blockchain is still at its infancy stage and it has a long way to go before it can be widely put in use. There are few challenges that have been raised by multiple researchers based on our review that need to be addressed. Some of these are discussed a little bit, being detailed in the following paragraphs below.

#### 4.2.1. Lack of Deep Understanding and Knowledge of the Blockchain Technology by Companies

The first challenge is to introduce the concept to the public, studies show that many people working on supply chain management are still having troubles to fully understand blockchain potentials [19,21,24,60,67]. The level of understanding of the technology can significantly impact the participants’ attitudes. According to the survey by Hackius and Petersen [24], participants who are more familiar with the concept and more experienced, tend to hold a more positive attitude to blockchain adoption. After analyzing some most recent pilot studies, Verhoeven et al. [23] also suggested that there is still lack of deep understanding of blockchain potential, as many companies tend to choose blockchain as solution before diagnosing company issues. For instance, Verhoeven et al. [23] suggested that, in Walmart’s pilot study of tracing mangoes, the tracing speed increased by blockchain should have been due to the elimination of the manual validation process rather than a change to an efficient platform. The mango pilot study also failed to present the importance of the immutability feature, as the records can be very important for fresh food quality. For example, the immutable records that include temperature and humidity can be the evidence for keeping food. Leong et al. [68] also suggested that different stages of the supply chain might have different requirements of technology adoption. Therefore, it is always necessary to have a comprehensive understanding of both costs and benefits of the technology to provide the right “remedy” to company problems; sometimes, other existing technologies can be better solutions [23,68].

#### 4.2.2. Technology Scalability Issue

The second challenge is blockchain scalability, which is also called “scalability trilemma” by the founder of Ethereum—Vitalik Buterin [69]. According to him, it is hard to achieve decentralization, scalability, and security at the same time; only two out of the three can be achieved at a point in time [69,79]. Bitcoin, for example, was designed to be decentralized and security with a compromising scalability.

Scalability determines how large the capacity the network can be. Currently, the smart contract platform—Ethereum, for example, can process 15 transactions per second, while other platforms, such as Visa, can process 45,000 transactions per second [80]. By operating the complicated mining process to validate transactions and keep all transaction copies in each node, blockchain can achieve high degree of decentralization and security; however, it can also cause a slow speed of validations, especially when a large number of transactions are happening. This means that high scalability can increase the security risks, while low scalability can cause transaction crowds and slow down the network.

Food supply chain tends to be vast with a large number of users exist in one transaction, the global food supply chain scale can reach approximately Petabyte per year by assumption according to [72]. Therefore, developers are still working to find a better solution to expend blockchain scalability while keeping high security and decentralization. Pearson et al. [72] assumed that blockchain is more likely to happen in niche areas in a food supply chain, where the blockchain potentials are necessarily needed, such as organic products, etc., due to the scalability issues. Leong et al. [68] also suggested that the different stages of the food supply chain might have different requirements for blockchain adoption, where the balance of the three features can be different.

#### 4.2.3. Possibilities of Raw Data Manipulation before Uploading to Blockchain

Although blockchain can provide a robust way to keep records, many studies have concerned raw data manipulation, for example by tempering with IoT sensors, it is hard to know whether the raw data in the first place are authentic [65,67]. It is also possible to make damage on products on purpose without notifying blockchain users [39]. Targeting on potential raw data manipulation, third parties, such as governments and certifications, can get involved in the blockchain network by making regular checks to ensure raw data authenticity [17,68]. Meanwhile, the immutable recording can be used as strategic tool to encourage suppliers to take responsibilities for their products and provide authentic information in the first place.

#### 4.2.4. It Is Hard to Require All Stakeholders within a Food Supply Chain to Adopt Blockchain

Rather than paper recording, blockchain requires all of the stakeholders to get involved, from raw material suppliers to customers along the multiple tiers of the supply chain. Stakeholders can register themselves as authorised users, which will allow them to upload information, to verify transactions, and to access past records. Customers as the end users will also have rights to require and check products’ history. Bringing all of the stakeholders on board can be beneficial for improving information transparency and efficiency; however, it also can be an obstacle due to the different level of awareness and infrastructures. For small and medium enterprises (SME) and developing countries, the blockchain implementing and infrastructure mentioning fee can be the barriers for adopting new innovations [60,68,69,72]. Kamilaris et al. [60] also found that most of the blockchain projects are located in developed counties. Therefore, it is important to make blockchain “SME friendly” which means easy to use, easy to deploy with low initial costs [68,72]. Perboli et al. [69] developed a blockchain model that was based on Hyperledger Fabric for SMEs and came to the conclusion that the implementation fee of blockchain can be highly sustainable and be paid back by saving on costs. They also suggested that replacing the system partially by blockchain is more reasonable than replacing the whole system.

#### 4.2.5. Regulations/Laws Need to Be Updated

Policies will be needed to protect users’ rights and trading secrets, as blockchain is an open database. Tse et al. [66] applied PEST analysis to examine the uncontrollable external environment, which includes political, economic, social, and technological factors for blockchain implementation. The paper suggested that governments could obtain supply chain information and reduce food risks by using blockchain. A few countries and authorities also showed their interests and supports on blockchain development [66]: China, for example, has published Blockchain White book and launched blockchain related projects [66]. ISO Blockchain (TC307) was also working on developing global blockchain standards [72]. There is still no strict blockchain policy in the food supply chain area so far. Leong et al. [68] and Pearson et al. [72] suggested that policies and rules need to be developed to protect users, including what data should be uploaded, who own the data, how to use and store the data, etc. Kamilaris et al. [60] proposed that a lack of policy could be the barrier for blockchain wide adoption. Therefore, from protecting users’ rights point of the view, it is difficult to invite all companies or people to use blockchain before some completed standards and policies are being launched.

## 5. Conclusions and Limitations

This paper presents a comprehensive review of blockchain based food supply chain. Most of the reviewed paper agreed on the promising benefits that blockchain may bring to the food supply chain. From building conceptual frameworks or analysing case studies, blockchain is found to be able to bring transparency, enhance information authenticity, and speed up food recall. Combining with current IoT technology, such as RFID, blockchain can further improve the efficiency of supply chain management and traceability system. Nevertheless, despite it drawing increasing attention from researchers, blockchain is in its infancy stage with many challenges. The challenges are waiting to be addressed before the technology can be put in use publicly. For technology adopters, such as food suppliers, there is a lack of deep understanding of the technology, which can compromise the benefits of blockchain. In another words, technology should be chosen for the issues rather than another way around. For technology developers, the low scalability is also an issue, which can cause a transaction crowd due to a large number of users and transactions in one food supply chain. For third parties, such as governments and organizations, there is no common admitted standard on blockchain adoption in food supply chain thus far. The development of policies can give protection to companies on their trading secrets and data storage. In this case, third parties are suggested to be more supportive in technology adoption, including technology education to the public, the development of certain policies and rules, and engagement with blockchain by pilot projects. In total, blockchain shows a significant potential that might address food crisis and bring a more trusted future on food security and quality.

This paper is one of the first to investigate how blockchain influences food supply chain specifically. The paper provides a fundamental and comprehensive understanding of blockchain and its potential impacts, which will not only be a useful guide for new researcher in relevant area, but can also provide some deeper insights for practitioners, such as company decision-makers. By identifying and analysing the most related papers, this work lays a solid ground for future research on this area and points out some research directions. This paper also gives technology adopters a better understanding of blockchain and explains to them some possible adoption challenges and reminds them to use blockchain wisely.

Despite the contributions put forth by this paper, we would like to point out some limitations and future research areas. First of all, the paper is based on 26 papers review, which might not be enough to prevent research bias. This is due the immaturity of blockchain technology; only several pilot studies with most of the theory-based articles are included. As there was big success of blockchain application in financial sector, the “hype” of blockchain is increasing and it gains a lot of attention. This can lead to potentially positive perspectives of blockchain rather than questioning the technology. In this case, future research can focus on blockchain implementation in the real world and provide more empirical evidence rather than theories. Secondly, food supply chain is vast and complicated. Different food products, such as fresh food, frozen food, agri-food, processed food, etc., may require difference supply chain. However, this review collects 26 food supply chain related papers without identifying particular products. Future research is possible to focus on specific food products, and present more precise findings.

## Figures and Tables

**Table 1:** Inclusion and Exclusion Criteria.

Inclusion	Exclusion
Published in English language	Published in other languages
Papers focus on food supply chain only	Papers focus on any industry rather than food supply chain industry
Published since 2008 to August 2019	Published before 2008
Papers focus on blockchain	Papers focus on other technologies
Peer review/conference papers, and grey articles	Business news
Management focus	Technique focus

**Table 2:** List of papers by content analysis.

Authors		[22]	[56]	[57]	[58]	[24]	[64]	[65]	[62]	[17]	[66]	[55]	[59]	[67]	[39]	[68]	[28]	[69]	[63]	[70]	[61]	[71]	[23]	[32]	[60]	[72]	[21]
Benefits	Transparency	✓	✓	✓	✓	✓		✓	✓	✓	✓	✓	✓	✓	✓	✓	✓	✓	✓	✓	✓		✓	✓	✓	✓	✓
	Information authenticity	✓	✓	✓	✓	✓			✓	✓	✓	✓	✓	✓	✓	✓		✓	✓		✓	✓	✓	✓	✓	✓	✓
	Sustainability				✓	✓		✓	✓		✓			✓	✓	✓		✓	✓	✓	✓		✓	✓	✓	✓	✓
	Efficiency	✓	✓	✓	✓	✓	✓	✓	✓	✓	✓		✓	✓	✓	✓	✓	✓	✓	✓	✓	✓	✓	✓	✓	✓	✓
Challenges	Lack of understanding				✓	✓								✓		✓				✓	✓		✓		✓		✓
	Immature technology				✓					✓	✓		✓		✓			✓			✓				✓	✓	✓
	Stakeholder cooperation				✓										✓	✓		✓			✓				✓	✓	
	Trade secrets				✓			✓					✓		✓	✓	✓				✓				✓	✓	✓
	Raw data manipulation				✓			✓							✓		✓										
